# Impact of the academic calendar cycle on survival outcome of injured patients: a retrospective cohort study at a community emergency department in Japan

**DOI:** 10.1186/s40560-019-0395-z

**Published:** 2019-08-01

**Authors:** Yuko Ono, Takeyasu Kakamu, Tokiya Ishida, Tetsu Sasaki, Shigeaki Inoue, Joji Kotani, Kazuaki Shinohara

**Affiliations:** 1 Department of Anesthesiology, Ohta General Hospital Foundation, Ohta Nishinouchi Hospital, 2-5-20 Nishinouchi, Koriyama, Fukushima 963-8558 Japan; 2 Department of Disaster and Emergency Medicine, Graduate School of Medicine, Kobe University, Kobe, Hyogo 650-0017 Japan; 3 Department of Hygiene and Preventive Medicine, School of Medicine, Fukushima Medical University, 1 Hikarigaoka, Fukushima, Fukushima 960-1295 Japan

**Keywords:** Academic cycle, Emergency surgery, Emergency endotracheal intubation, July phenomenon, Trauma, Seasonal effects

## Abstract

**Background:**

Commencement of a new academic cycle is presumed to be associated with poor patient outcomes. However, supportive evidence is limited for trauma patients treated in under-resourced hospitals, especially those who require specialized interventions and with little physiological reserve. We examined whether a new academic cycle affects the survival outcomes of injured patients in a typical Japanese teaching hospital.

**Methods:**

This historical cohort study was conducted at a Japanese community emergency department (ED). All injured patients brought to the ED from April 2002 to March 2018 were included in the analysis. The primary exposure was presentation to the ED during the first quartile of the academic cycle (April–June). The primary outcome measure was the hospital mortality rate.

**Results:**

Of the 20,945 eligible patients, 5282 (25.2%) were admitted during the first quartile. In the univariable analysis, the hospital mortality rate was similar between patients admitted during the first quartile of the academic year and those admitted during the remaining quartiles (4.1% vs. 4.4%, respectively; odds ratio [OR], 0.931; 95% confidence interval [CI] 0.796–1.088). After adjusting for the potential confounding factors of the injury severity score, age, sex, Glasgow coma scale score, systolic blood pressure, trauma etiology (blunt or penetrating), and admission phase (2002–2005, 2006–2009, 2010–2013, and 2014–2018), no statistically significant association was present between first-quartile admission and trauma death (adjusted OR 0.980; 95% CI 0.748–1.284). Likewise, when patients were subgrouped according to age of > 55 years, injury severity score of > 15, Glasgow coma scale score of < 9, systolic blood pressure of < 90 mmHg, requirement for doctor car system dispatches, emergency operation, emergency endotracheal intubation, and weekend and night presentation, no significant associations were present between first-quartile admission and hospital mortality in both the univariable and multivariable analysis.

**Conclusions:**

At a community hospital in Japan, admission at the beginning of the academic year was not associated with an increased risk of hospital mortality among trauma patients, even those requiring specialized interventions and with little physiological reserve. Our results support the uniformity of trauma care provision throughout the academic cycle in a typical Japanese trauma system.

**Electronic supplementary material:**

The online version of this article (10.1186/s40560-019-0395-z) contains supplementary material, which is available to authorized users.

## Background

Traumatic injury is a major healthcare concern worldwide. Trauma is the leading cause of death and disability among young people and places a tremendous economic burden on society [1, 2]. Trauma is the representative example of an unplanned critical condition requiring rapid diagnosis and aggressive intervention. Inadequate evaluation and care is known to lead to increased mortality in this high-risk condition [3, 4].

In teaching hospitals, a large transition takes place at the beginning of the academic year. In such seasons, many new and inexperienced healthcare providers such as residents, clinical fellows, new graduate nurses, and technicians begin caring for patients. The performance of experienced healthcare professionals may also temporarily decline after transfer to a new workplace because considerable time will be required to become accustomed to local systems and hospital rules. Therefore, any compromise in the care of injured patients may be likely to have a more pronounced impact at the beginning of the academic year.

However, previous reports have shown that if injured patients are treated at a level I trauma center in a mature trauma care system [5], the mortality rates of patients presenting at the beginning of the academic cycle are not higher than those of patients presenting during the rest of the academic year [6–10]. A level I trauma center [5] has the highest concentration of medical resources, and direct supervision of experienced house staff is likely to be available 24 h a day.

Unfortunately, such specialized trauma care has not yet been implemented everywhere in Japan. For example, most Japanese community hospitals, including our own, do not comply with the American College of Surgeons standards for a level I [5], or even a level II, trauma center [5]. Medical staff shortage is one of the most serious problems, especially in the provinces [11]. In such settings, a significant amount of patient care is provided by the resident or new house staff without sufficient supervision. To the best of our knowledge, no studies have been performed to examine the effect of the academic cycle on trauma outcomes in such under-resourced hospitals. Staff turnover at the beginning of the academic year may be more distinguished if injured patients require specialized interventions and have little physiological reserve. We also considered that injured patients who present during the night and weekend may be more susceptible to turnover effects because direct supervision of experienced staff is even less available during such periods. However, past studies have not fully clarified the effect of the academic calendar cycle on these important trauma subsets [6–10].

Therefore, we analyzed patients in a representative under-resourced hospital in a developing trauma care system to determine whether the outcomes of injured patients, especially in the above-mentioned subsets, differ significantly at the beginning versus the rest of the academic year cycle.

## Methods

### Study design and setting

This retrospective cohort study was conducted at a community hospital in a provincial Japanese city. The hospital serves both as a teaching facility and as a referral trauma center for a population of 538,000 inhabitants. Annually, the hospital receives > 5,500 ambulances and > 1300 trauma patients with injuries of varying severity from areas within a 50-km radius. Similar to most Japanese hospitals, the major changeover time for new house staff in the hospital is 1 April each year. Major staff changeover also takes place among emergency lifesaving technicians during the same period. Therefore, many new health care professionals, including emergency department (ED) physicians, trauma surgeons, anesthesiologists, junior and senior residents, nurses, radiology technicians, and emergency lifesaving technicians, begin providing trauma care in a new work environment in April.

In our hospital, injured patients are initially treated by a trauma resuscitation team that consists of attending ED physicians, emergency medicine residents, post-graduate year 1 or 2 junior residents, nurses, and radiology technicians. Attending and senior resident level trauma surgeons, anesthesiologists, and interventional radiologists are not always present with the injured patient in the ED, but they immediately respond from within the hospital and are actively involved in the resuscitation and all subsequent care during weekday business hours. If injured patients require emergency surgery or transarterial embolization during the weekend or nighttime hours, these specialists respond from outside the hospital. In most cases, the response time (time elapsed from call to presence at the ED) during off-hours is approximately 30 min. To expedite the collaboration between prehospital emergency lifesaving technicians, the hospital runs a prehospital emergency medical unit (doctor car system) consisting of a trained ambulance driver, a nurse, a senior ED physician, and a resident. This physician-delivery system is dispatched to the scene following a request by the on-scene emergency lifesaving technicians or regional medical control center.

### Participants and data sources

The review board at Ohta Nishinouchi Hospital approved this study on 18 June 2018 (Approval No. 3060). The study included all trauma patients brought to the ED from 1 April 2002 to 31 March 2018. The exclusion criteria were patients who received ongoing cardiopulmonary resuscitation on initial contact and patients who were transported from other facilities. The data were collected from a hospital-based electronic trauma database. This database captures each patient’s age, sex, initial vital signs, prehospital time (time from emergency call to ED arrival), requirements for emergency endotracheal intubation (ETI) and emergency surgery, preoperative time (time from ED arrival to operating room), and hospital survival. This database also records the trauma etiology (blunt or penetrating); injury severity determined by the injury severity score (ISS) [12]; and the revised trauma score (RTS) [13], a weighted physiological scoring system. These parameters were scored without delay by one of the authors (K.S.). The ISS was based on information obtained by physical examination and by radiological and operative findings. The RTS was based on the initially recorded vital signs. Our department maintains a rigorous peer review process: An ED director at our hospital (K.S.) checks all medical records to verify the completeness and reliability of these data at the earliest possible opportunity.

### Exposures and outcome measurement

The primary exposure was presentation to the ED during the first quartile of the academic cycle (April–June). The primary outcome measure was the hospital mortality rate of injured patients. We examined quarters because the turnover effect is not only limited to the first month (April) of the academic year. Analyses examining quarters of admission also gives more power to detect statistically significant differences in hospital mortality. Past studies that were conducted in intensive care unit settings [14] or that examined the impact of the academic calendar cycle on patients undergoing high-risk surgical procedures [15, 16] also employed a similar survey strategy and measurement.

### Statistical analysis

First, the differences in the baseline clinical characteristics of trauma patients admitted to our ED during the first versus second to fourth quartile of the academic cycle were evaluated. Differences in continuous variables between the two groups were compared using Student’s *t* test after first verifying the normal distribution of the data using the Kolmogorov–Smirnov test; otherwise, the Mann–Whitney *U* test was used. Differences in categorical variables between the two groups were compared using the chi-squared test followed by residual analysis. The crude odds ratio (OR) was then calculated to estimate the relative risk of death of injured patients brought to the ED during the first quartile using a 2 × 2 contingency table. The chi-squared test was used to produce the *p* values.

A multivariate logistic regression analysis was used to adjust for the potential confounders of ISS, age, sex, systolic blood pressure (SBP), Glasgow coma scale (GCS) score, trauma etiology (blunt or penetrating), and admission phase, which yielded an adjusted OR for death after first-quartile presentation as the primary exposure. A set of these variables was chosen a priori based on previous reports [12, 13, 17–20] and biological plausibility. We divided the patients’ SBP into four groups (1–49, 50–75, 76–89, and > 89 mmHg) and the GCS score into five groups (3, 4–5, 6–8, 9–12, and ≥ 13) based on the scoring system of the RTS [13] to optimize the model fit of multivariable regression. Our study period was quite long, and during this time a standardized trauma education program (Japan Advanced Trauma Evaluation and Care™) was introduced throughout Japan [19], including at our facility. Because the introduction of this trauma education program could affect trauma care and outcomes, we separated our sample into four phases (2002–2005, 2006–2009, 2010–2013, and 2014–2018) and considered each phase as a possible confounder as described by Hondo et al. [20]. Our hospital resources, however, including the trauma response and on-call system described in the “Study design and setting” section, remained relatively unchanged during the study period. The annual number of physicians (post-graduate year 1 or 2 residents, ED physicians, and physicians with other specialties) and nurses during the study period is shown in Additional file 1: Figure S1. The categorized intervals of an SBP of > 89 mmHg, GCS score of ≥ 13, and admission phase of 2002–2005 were set as references when establishing the logistic regression model of hospital mortality.

In all logistic regression models, a variance-inflation factor was used to detect multicollinearity. The model’s discrimination abilities were confirmed with the c statistic.

### Subanalysis

Differences in hospital mortality between two groups were also assessed in prespecified subgroups of patients. Multivariate logistic regression analyses were repeated in the subgroup of patients with an ISS of > 15, age of > 55 years, SBP of < 90 mmHg, and GCS score of < 9 as well as in the subgroups of patients who required doctor car dispatch, required emergency ETI and emergency surgery, and presented during off-hours. For consistency with our own studies and those of other researchers [21–24], off-hours were defined as the period from 6:01 pm to 8:00 am on weekdays plus the entire weekend.

To further clarify the turnover effect on trauma outcomes, analyses were performed to compare the hospital mortality rate of patients admitted in April with those admitted from May to March. We also compared the hospital mortality for each month and each quartile to reveal seasonal variation.

Finally, because early operative control of hemorrhage is vital in injured patients [25] and the preoperative time was considered to be an important parameter of trauma care in many previous studies [26–28], we compared the time from ED arrival to the operating room (preoperative time) between patients who underwent emergency surgery during the first and remaining quartiles of the academic cycle. To evaluate prehospital trauma care performance, we also compared the time from the emergency call to ED arrival (prehospital time) between these two groups.

All statistical analyses were performed using SPSS Statistics for Windows, version 22.0 (IBM Corp., Armonk, NY). A *p* value of < 0.05 was considered to indicate statistical significance.

### Power analysis

During the planning phase of this study, a power analysis was performed using Power and Sample Size Calculation version 3.1.2 (Department of Biostatistics, Vanderbilt University School of Medicine, Nashville, TN). To calculate the sample size for the current study, we estimated that the incidence of trauma-related death in the first quartile would be approximately 5%. This assumption was based on a similar study conducted in the USA [6–10] and our preliminary observation (2016–2017 pilot data). Assuming a 1:3 ratio of injured patients presenting during the first quartile and the remaining quartiles, 4589 and 13,767 patients (total of 18,356) were required to obtain a 1.0% hospital mortality difference between two groups at a two-tailed α of 0.05 and power of 0.80. Because approximately 1300 trauma patients were brought to our hospital annually, we decided to include trauma patients for 16 years to allow for a 10% exclusion rate.

## Results

**Fig. 1:**
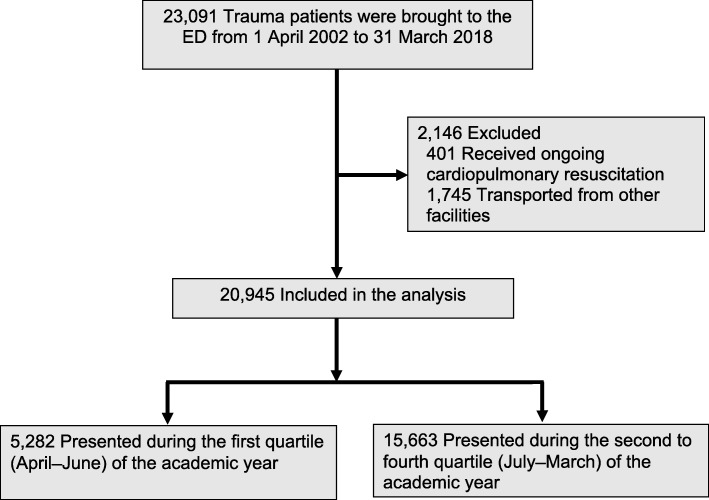
Flow chart showing the selection process for injured patients included in primary analysis. In Japan, the academic year cycle typically begins on 1 April each year. The first academic quartile therefore corresponds to April to June. *ED* emergency department

During the study period, 23,091 trauma patients were brought to the ED (Fig. 1). Of these, we excluded 401 patients who received ongoing cardiopulmonary resuscitation and 1745 patients who were transported from other facilities. The remaining 20,945 patients were included in the analysis. Of these patients, 5282 (25.2%) were admitted to our ED during the first quartile and 15,663 (74.8%) were admitted during the second to fourth quartile (Fig. 1). Complete records were available for all patients, and no data were missing from the analyses.

Although statistically significant differences in age (47.0 ± 27.2 vs. 49.4 ± 26.6 years, *p* < 0.001), male sex (60.6% vs. 58.1%, *p* = 0.002), and off-hour presentation (61.5% vs. 58.6%, *p* < 0.001) were observed between the first quartile and remaining quartiles, the magnitude of the differences was clinically small (Table 1). Other clinical characteristics, including the admission phase, trauma etiology, anatomical and physiological severity scale scores, doctor car dispatches, and requirements for emergency surgery and emergency ETI, were similar between the two groups (Table 1).

**Table 1:** Clinical characteristics of injured patients: first academic quartile (April–June) versus second to fourth quartile (July–March)

	All (*n* = 20,945)	April–June^a^ (*n* = 5,282)	July–March^b^ (*n* = 15,663)	*p*
Age, years	48.8 ± 26.8	47.0 ± 27.2	49.4 ± 26.6	< 0.001
Male	12,304 (58.7)	3,200 (60.6)	9,104 (58.1)	0.002
Admission phase				0.443
2002–2005	5687 (27.2)	1426 (27.0)	4261 (27.2)	
2006–2009	5477 (26.1)	1412 (26.7)	4065 (26.0)	
2010–2013	4984 (23.8)	1271 (24.1)	3713 (23.7)	
2014–2018	4797 (22.9)	1173 (22.2)	3624 (23.1)	
Trauma etiology				0.359
Blunt	19,912 (95.1)	5009 (94.8)	14,903 (95.1)	
Penetrating	1033 (4.9)	273 (5.2)	760 (4.9)	
Anatomical severity				
ISS	7.2 ± 10.9	7.2 ± 11.3	7.3 ± 10.8	0.138
Physiological severity				
GCS score				0.205
13–15	19,366 (92.5)	4884 (92.5)	14,482 (92.5)	
9–12	527 (2.5)	136 (2.6)	391 (2.5)	
6–8	287 (1.4)	86 (1.6)	201 (1.3)	
4–5	157 (0.7)	33 (0.6)	124 (0.8)	
3	608 (2.9)	143 (2.7)	465 (3.0)	
SBP, mmHg				0.686
> 89	20,091 (95.9)	5076 (96.1)	15,015 (95.9)	
76–89	213 (1.0)	47 (0.9)	166 (1.1)	
50–75	205 (1.0)	50 (0.9)	155 (1.0)	
1–49	436 (2.1)	109 (2.1)	327 (2.1)	
Respiratory rate, breaths/min				0.971
> 29	19,897 (95.0)	5023 (95.1)	14,874 (95.0)	
10–29	592 (2.8)	147 (2.8)	445 (2.8)	
6–9	50 (0.2)	14 (0.3)	36 (0.2)	
1–5	9 (0.04)	2 (0.03)	7 (0.04)	
0	397 (1.9)	96 (1.8)	301 (1.9)	
RTS	7.55 ± 1.21	7.56 ± 1.19	7.55 ± 1.22	0.768
Doctor car system dispatched	2357 (11.3)	618 (11.7)	1739 (11.1)	0.235
Off-hours presentation^b^	12,424 (59.3)	3248 (61.5)	9176 (58.6)	< 0.001
Emergency surgery	3007 (14.4)	757 (14.3)	2250 (14.4)	0.952
Emergency ETI	1253 (6.0)	315 (6.0)	938 (6.0)	0.947

Data are expressed as mean ± standard deviation, *n* (%), or median (interquartile range)

*ETI* endotracheal intubation, *GCS* Glasgow coma scale, *ISS* injury severity score, *RTS* revised trauma score, *SBP* systolic blood pressure

^a^In Japan, the academic cycle typically begins on 1 April each year. The first academic quartile therefore corresponds to April to June

^b^6:01 pm to 8:00 am on weekdays plus all weekend hours

In the univariable analysis, the hospital mortality among patients admitted during the first quartile of the academic year was similar to that among patients admitted during the second to fourth quartiles (4.1% vs. 4.4%; OR 0.931; 95% confidence interval [CI] 0.796–1.088) (Table 2). After adjusting for the potential confounding factors of age, sex, ISS, GCS score, SBP, trauma etiology, and admission phase, no statistically significant association was present between first-quartile admission and trauma death (adjusted OR 0.980; 95% CI 0.748–1.284) (Table 2).

**Table 2:** Logistic regression models for hospital mortality in injured patients: first academic quartile (April–June) versus second to fourth quartile (July–March)

	Patients, *n*			Univariable analysis		Multivariable analysis^b^	
	Total	Dead	Mortality rate (%)	OR (95% CI)	*p*	Adjusted OR (95% CI)	*p*
Full cohort (N = 20,945)							
April–June^a^ (reference)	5282	215	4.1	0.931 (0.796–1.088)	0.368	0.980 (0.748–1.284)	0.885
July–March	15,663	683	4.4				

The reference set was the group of patients admitted to the emergency department during the first academic quartile (in Japan, April–June)

*CI* confidence interval, *OR* odds ratio

^a^In Japan, the academic cycle typically begins on 1 April each year. The first academic quartile therefore corresponds to April to June

^b^Adjustment for the potential confounders of age, sex, injury severity score, Glasgow coma scale score, systolic blood pressure, trauma etiology (blunt or penetrating), and admission phase (2002–2005, 2006–2009, 2010–2013, and 2014–2018)

When patients were subgrouped according to an age of > 55 years, ISS of > 15, GCS score of < 9, SBP of < 90 mmHg, requirement for doctor car system dispatches, emergency surgery, emergency ETI, and off-hour presentation, there remained no significant associations between first-quartile admission and hospital mortality in either the univariable or multivariable analysis (Table 3).

**Table 3:** Subgroup analysis of hospital mortality in injured patients across academic cycles

	No of patients			Univariable analysis		Multivariable analysis^a^	
	Total	Dead	Mortality rate (%)	OR (95% CI)	*p*	Adjusted OR (95% CI)	*p*
By physiological reserve							
Age of > 55 years (*n* = 9693)							
April–June (reference)^b^	2319	131	5.6	0.906 (0.742–1.107)	0.335	0.990 (0.724–1.355)	0.952
July–March	7374	457	6.2				
ISS of > 15 (*n* = 2974)							
April–June (reference)^b^	741	184	24.8	0.907 (0.749–1.099)	0.319	0.912 (0.677–1.228)	0.545
July–March	2233	596	26.7				
GCS score of < 9 (*N* = 1052)							
April–June (reference)^b^	648	153	23.6	0.837 (0.629–1.112)	0.219	0.838 (0.560–1.253)^c^	0.390
July–March	404	109	27.0				
SBP of < 90 mmHg (*n* = 854)							
April–June (reference)^b^	206	137	66.5	1.231 (0.885–1.712)	0.216	1.693 (0.968–2.959)^d^	0.065
July–March	648	400	61.7				
By specialized intervention							
Doctor car system dispatched (*n* = 2357)							
April–June (reference)^b^	618	121	19.6	0.910 (0.723–1.145)	0.422	1.010 (0.681–1.499)	0.961
July–March	1739	367	21.1				
Emergency surgery (*n* = 3007)							
April–June (Reference)^b^	757	43	5.7	0.944 (0.663–1.344)	0.790	0.918 (0.565–1.493)	0.731
July–March	2250	135	6.0				
Emergency ETI (*n* = 1253)							
April–June (reference)^b^	315	178	56.5	0.909 (0.702–1.176)	0.466	0.999 (0.699–1.426)	0.994
July–March	938	552	58.8				
Off-hours presentation^e^ (*n* = 12,424)							
April–June (reference)^b^	3248	146	4.5	0.872 (0.721–1.054)	0.173	1.020 (0.735–1.417)	0.904
July–March	9176	470	5.1				
By different definition							
April vs. remaining months (*n* = 20,945)							
April (reference)^b^	1744	77	4.4	1.034 (0.814–1.313)	0.764	0.886 (0.576–1.361)	0.580
May–March	19,201	821	4.3				

*CI* confidence interval, *ED* emergency department, *ETI* endotracheal intubation, *GCS* Glasgow coma scale, *ISS* injury severity score, *OR* odds ratio, *SBP* systolic blood pressure

^a^Adjustment for the potential confounders of age, sex, ISS, GCS score, SBP, trauma etiology (blunt or penetrating), and admission phase (2002–2005, 2006–2009, 2010–2013, and 2014–2018) unless otherwise indicated

^b^In Japan, the academic cycle typically begins on 1 April each year. The first academic quartile therefore corresponds to April to June

^c^Categorized GCS score (3, 4–5, 6–8, 9–12, and ≥ 13) was removed from the set of explanatory variables because of the model validation

^d^Categorized SBP (1–49, 50–75, 76–89, and > 89 mmHg) was removed from the set of explanatory variables because of the model validation

^e^6:01 pm to 8:00 am on weekdays plus all weekend hours

**Fig. 2:**
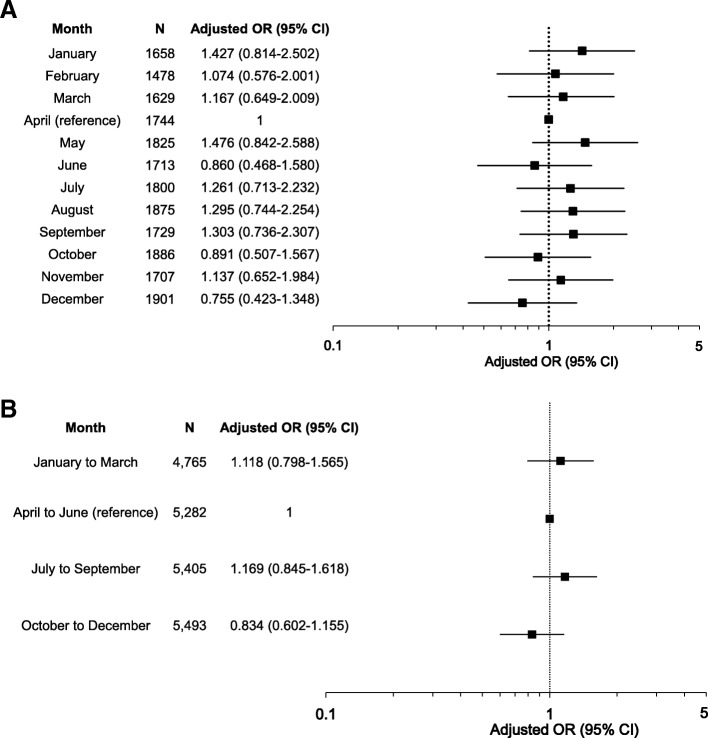
Adjusted OR with 95% CI for hospital mortality by (**a**) month and (**b**) academic quartile. Adjustment for the potential confounders of age, sex, ISS, GCS score, SBP, trauma etiology (blunt or penetrating), and admission phase (2002–2005, 2006–2009, 2010–2013, and 2014–2018). **a** The reference set was the group of patients admitted to the ED during first month (in Japan, April) of the academic year. **b** The reference set was the group of patients admitted to the ED during the first quartile (in Japan, April–June) of the academic year. *CI* confidence interval, *ED* emergency department, *GCS* Glasgow coma scale, *ISS* injury severity score, *OR* odds ratio, *SBP* systolic blood pressure

The subanalysis comparing survival between patients admitted in April versus the remaining duration of the academic cycle also showed no statistical significance (Table 3). Moreover, no difference in the odds of death was found when months and quartiles of admission were evaluated separately (Fig. 2a, b).

**Fig. 3:**
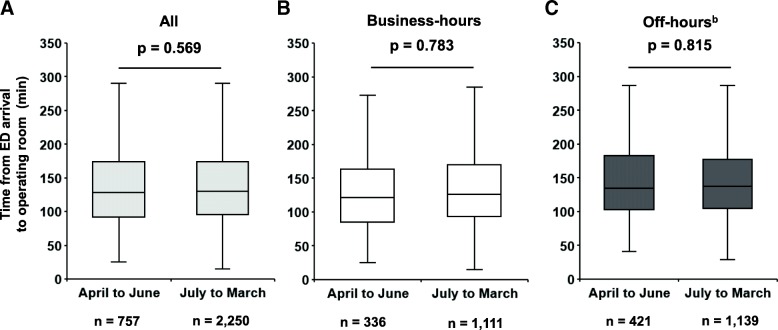
Preoperative time in injured patients requiring emergency surgery: first versus second to fourth academic quartile^a^. Box plots of time from ED arrival to operating room in injured patients who underwent emergency surgery. **a** All injured patients, **b** patients who presented during business hours, and **c** patients who presented during off-hours^b^. The solid line within the box represents the median, the box represents the 25th and 75th percentiles, and the whiskers represent the lower and upper extremes. The *p* value was derived from the Mann–Whitney U-test. ^a^In Japan, the academic cycle typically begins on 1 April each year. The first academic quartile therefore corresponds to April to June, and the second to fourth quartile corresponds to July to March. ^b^Off-hours are defined as the period from 6:01 pm to 8:00 am on weekdays plus the entire weekend. *ED* emergency department

Finally, among 3007 patients who underwent emergency surgery, the time from ED arrival to operating room arrival was similar between the first academic quartile group and the second to fourth quartile group (Fig. 3a). A similar trend remained after stratification by the presentation time (business-hours or off-hours) (Fig. 3b, c).

**Fig. 4:**
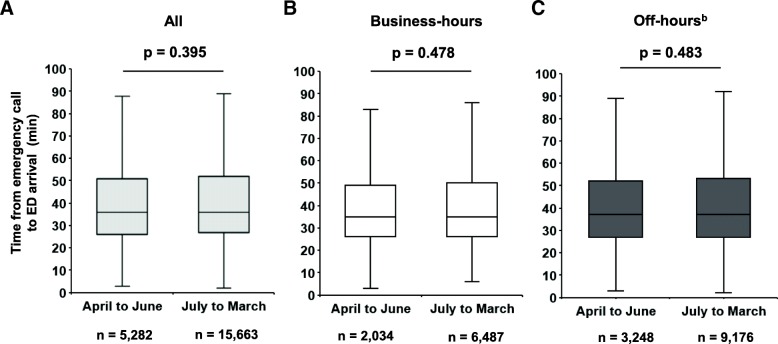
Prehospital time in injured patients: first versus second to fourth academic quartile^a^. Box plots of time from emergency call to ED arrival in (**a**) all injured patients, **b** patients who presented during business hours, and **c** patients who presented during off-hours^b^. The solid line within the box represents the median, the box represents the 25th and 75th percentiles, and the whiskers represent the lower and upper extremes. The *p* value was derived from the Mann–Whitney *U* test. ^a^In Japan, the academic cycle typically begins on 1 April each year. The first academic quartile therefore corresponds to April to June, and the second to fourth quartile corresponds to July to March. ^b^Off-hours are defined as the period from 6:01 pm to 8:00 am on weekdays plus the entire weekend. ED: emergency department

The prehospital time (time from emergency call to ED arrival) was also similar between these two groups (Fig. 4a, all; Fig. 4b, business hours; and Fig. 4c, off-hours).

We detected no multicollinearity (variance inflation factor of < 3.0 for each explanatory variable in each model), and the c statistic showed acceptable discrimination for the logistic models (all > 0.8).

## Discussion

In this retrospective cohort study, we found that admission to the ED of a Japanese community teaching hospital from April through June was not associated with an increased risk of hospital mortality. There were also no significant differences in hospital mortality when months and quartiles of admission were evaluated separately. Moreover, the results were consistent when patients were subgrouped according to their physiological reserves (age of > 55 years, ISS of > 15, SBP of < 90 mmHg, and GCS score of < 9), requirement for specialized care (doctor car system dispatch, emergency surgery, and emergency ETI), and admission during the weekend and night. Collectively, these results suggest that the beginning of the academic quarter is no more perilous to patients than any other time of the year in a typical Japanese trauma system.

In this analysis of more than 20,000 injured patients, the survival outcome was similar between trauma patients admitted to our ED during the first quartile and those admitted during the second to fourth quartile of the academic cycle. The sample size was large enough to attain a 1.0% hospital mortality difference between these two groups. In addition, there were no missing data because we used a prespecified ED database and our department has a rigorous peer review process supervised by its director. We therefore believe that our study reliably indicates the homogeneity of trauma care throughout the academic calendar cycle. Our findings are also in line with those of earlier observations in level I trauma centers in the USA. For example, in a retrospective observational study of a North American ED, Schroeppel et al. [8] analyzed 14,559 patients by quarter during a 5-year period and found no variation in quality-of-care indicators including the hospital mortality rate, ventilator support days, intensive care unit days, and times in the resuscitation room. In a retrospective analysis of 8151 trauma patients at two academic level I trauma centers in the USA, Inaba et al. [9] reported that admission at the beginning of the academic year was associated with an increased risk of errors and preventable complications, but these errors did not impact hospital mortality. Our data corroborate and expand these findings by using a larger trauma patient sample in a much less-resourced practical setting.

Previous studies did not fully assess the associations between the academic cycle and outcome of trauma patients who required specialized care and had unstable vital signs [6–10]. Staff changeover may have a more pronounced impact on such patients because they require more complex and refined procedures. We examined these relationships in the present study and found that the hospital mortality rate was not increased in these subsets of patients in the first quartile of the academic year. Moreover, among injured patients who underwent emergency surgery, almost no preoperative time variation was found between the first and second to fourth quartiles of the academic cycle. These results further support the uniformity of care provision in injured patients who required subspecialty intervention across the year. At our institution, trauma patients, especially those in the above-mentioned subsets, are treated by a trauma resuscitation team consisting of attending-level ED physicians, residents at various levels of training, and paramedical staff such as nurses and radiology technicians. Attending and senior resident levels of surgeons, anesthesiologists, and interventional radiologists readily respond to trauma activation and actively participate in the resuscitation and subsequent care. Such an interdisciplinary collaboration and organized team approach [29–32] to the diagnosis and resuscitation of trauma patients might work in a complementary fashion and prevent the detrimental effects of major staff turnover. Additionally, such a multidisciplinary team approach is known to be associated with improved care provision [29–32]. Our data were also consistent with previous studies that were conducted in other settings and examined other critical patient populations requiring multidisciplinary care [14–16, 33].

Similar to most medical institutions, staffing levels dramatically decrease during off-hours in our hospital. At such times, experienced doctors in supervisory roles and consultants in subspecialties are less available [21–24]. Although the detrimental effects of staff changeover may have been more evident during such times, neither the hospital mortality rate nor the preoperative time was increased in these subsets of patients in the beginning of the academic year. These results further strengthen our conclusion that there is no variation in trauma care provision across the academic calendar cycle.

This study has several limitations. The first is the crude estimate of patients’ outcomes. While the hospital mortality rate is an important endpoint, we did not account for other key outcomes such as the duration of the ED stay, preventable errors and complications, or cost-effectiveness. Instead, our database included the prehospital and preoperative time as a care parameter, which previous reports have not examined [6–10]. Our data showed almost no variation in these parameters between the first and second to fourth quartiles of the academic cycle, supporting the uniformity of prehospital and preoperative trauma care quality throughout the academic cycle. Second, while our ED is typical of a Japanese community ED, as with any single-center study, it may not be possible to extrapolate our findings to other medical institutions, especially those in other countries. Third, similar to previous studies [6–10, 14–16, 33], our database did not capture information regarding how many physicians, senior and junior residents, nurses, emergency lifesaving technicians, or other paramedical staff were actually transferred. Our database also did not include information regarding how many physicians, nurses, and other healthcare professionals were actually involved in trauma care. Although adjustments were made for previously known confounding factors [12, 13, 17–20] using a logistic regression model, these and other unmeasured factors may have confounded our results, as with any observational study.

Despite these limitations, this study has revealed the effects of major staff transition on injured patients at a community hospital in Japan. We believe that this study represents the current state of trauma care in similar under-resourced hospitals and supports the acceptance of the current academic cycle in a high-risk patient subset.

## Conclusions

At a community hospital in Japan that does not comply with the American College of Surgeons standards for a level II trauma center, major staff changeover at the beginning of the academic year was not associated with increased hospital mortality among injured patients. These results were consistent in the subgroups of patients who had unstable vital sings, required specialized care, and were admitted during the weekend and night. Our results support the homogeneity of trauma care across the academic cycle in a typical Japanese trauma system.

## Additional file

Additional file 1: **Figure S1.** (A) Annual number of post-graduate year 1 or 2 residents, emergency department physicians, and physicians with other specialties during the study period. (B) Annual number of nurses during the study period. (PDF 18 kb)

## Data Availability

All data relevant to the study are included in this published article. Further datasets analyzed during the study are available from the corresponding author on reasonable request.

## Abbreviations

CI: Confidence interval
ED: Emergency department
ETI: Endotracheal intubation
GCS: Glasgow coma scale
ISS: Injury severity score
OR: Odds ratio
RTS: Revised trauma score
SBP: Systolic blood pressure
