# Supplementary material for: Impact of the academic calendar cycle on survival outcome of injured patients: a retrospective cohort study at a community emergency department in Japan
Source: J Intensive Care. 2019 Aug 1;7:39. doi: 10.1186/s40560-019-0395-z (PMC6669975; doi:10.1186/s40560-019-0395-z)

Figure S1A.
Annual number of post-graduate year 1 or 2 residents, ED physicians, and physicians with other specialties during the study period.

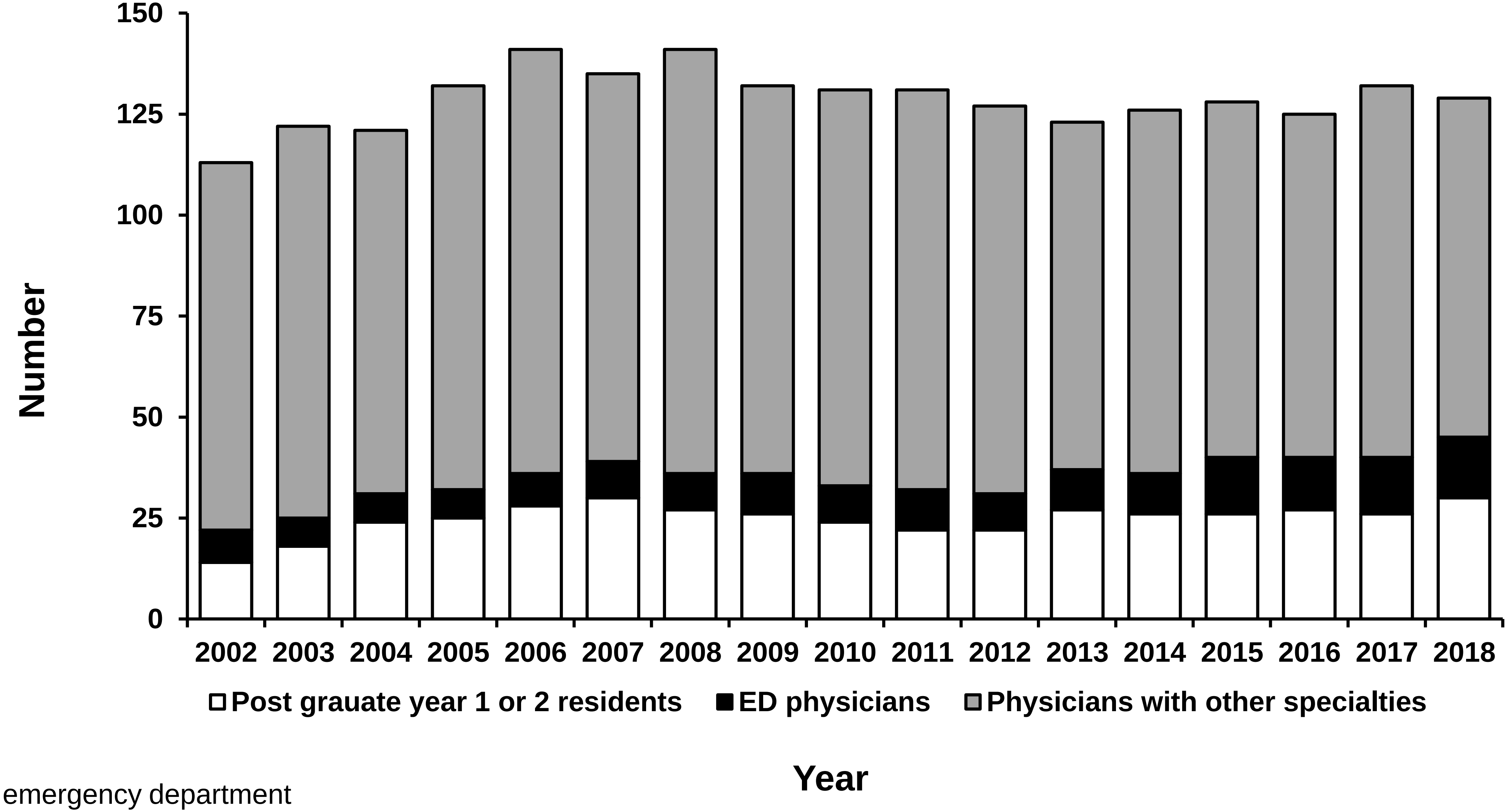

ED: emergency department

Figure S1B.
Annual number of nurses during the study period.

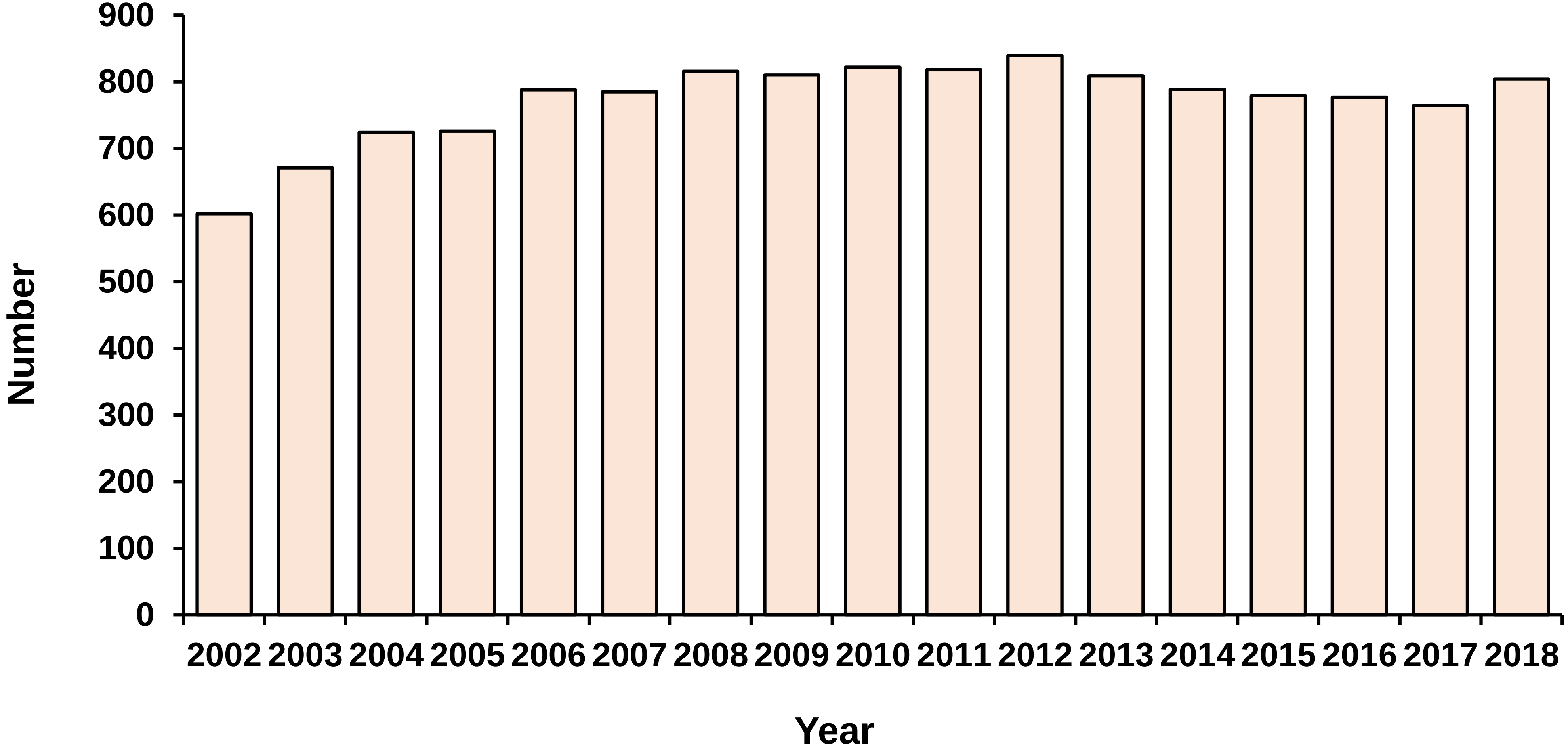

Supplement: Supplementary file 1 — Figure S1. (A) Annual number of post-graduate year 1 or 2 residents, emergency department physicians, and physicians with other specialties during the study period. (B) Annual number of nurses during the study period. (PDF 18 kb) [file 40560_2019_395_MOESM1_ESM.pdf]
